# Impact of depression on the quality of sleep and immune functions in patients with coronary artery disease

**DOI:** 10.1136/gpsych-2022-100918

**Published:** 2022-12-27

**Authors:** Liqiang Cai, Lili Wei, Jiashu Yao, Yanhua Qin, Yafeng You, Luoyi Xu, Jinsong Tang, Wei Chen

**Affiliations:** Department of Psychiatry, Sir Run Run Shaw Hospital, Zhejiang University School of Medicine, Hangzhou, Zhejiang, China

**Keywords:** depressive disorder, major, psychosomatic medicine

## Abstract

**Background:**

The risk of major depressive disorder (MDD) and insomnia is higher in patients with coronary heart disease (CHD) than in the general population. In addition, immune inflammation may be a shared aetiological factor for mental disorders and CHD. However, it is unclear whether MDD is associated with poor sleep quality and cell-mediated immune function in patients with CHD.

**Aims:**

This study investigated the impact of depression on sleep quality and cell-mediated immune functions in patients with CHD and examined discriminative factors in patients with CHD with and without MDD.

**Methods:**

This cross-sectional retrospective study was conducted at the Zhejiang University School of Medicine affiliated with Sir Run Run Shaw Hospital. The study population consisted of 84 patients with CHD assigned to two groups based on their Hamilton Depression Rating Scale (HAMD) score (CHD with MDD (HAMD score of ≥10) vs without MDD). Subjective sleep quality, systemic inflammatory response and cell-mediated immune functions were assessed in patients with CHD with (n=50) and without (n=34) MDD using the Pittsburgh Sleep Quality Index (PSQI), routine blood tests and flow cytometry. The relationships between variables were ascertained using Pearson’s product–moment, and linear discriminant analysis was used to explore the discriminative factors between groups.

**Results:**

Patients with CHD with MDD had significantly poorer sleep quality than those without MDD (Z=−6.864, p<0.001). The Systemic Inflammation Index (SII) and CD4^+^/CD8^+^ T-cell ratios were higher in patients with CHD with MDD than in those without MDD (Z=−3.249, p=0.001). Patients with CHD with MDD had fewer CD3^+^CD8^+^ and CD3^+^ T cells (Z=3.422, p=0.001) than those without MDD (t=2.032, p=0.045). Furthermore, patients with CHD with MDD may be differentiated from those without MDD using the PSQI, SII and T-cell levels, as these variables correctly classified the depressed and non-depressed groups with an accuracy of 96.4%.

**Conclusions:**

MDD may be responsible for poor sleep quality, increased cell-mediated immunity and SII in patients with CHD, which are discriminative factors for CHD in the depressive state. Clinicians should be aware of these interactions, as treatment for depressive symptoms may also improve CHD prognosis.

**WHAT IS ALREADY KNOWN ON THIS TOPIC**

- Major depressive disorder (MDD), poor sleep quality and heart disease are epidemiologically linked; however, their correlation is not well understood. Furthermore, it is unclear whether depression is associated with poor sleep quality and cell-mediated immune function in coronary heart disease (CHD).

**WHAT THIS STUDY ADDS**

- MDDs may be responsible for poor sleep quality, increased cell-mediated immunity and Systemic Inflammation Index in patients with CHD, which are discriminative factors for CHD in the depressive state.

**HOW THIS STUDY MIGHT AFFECT RESEARCH, PRACTICE OR POLICY**

- The study results may inform potential strategies for screening, prevention or treatment of MDD in CHD. Clinicians should be aware of these identified discriminative factors because treating insomnia and depression may also improve CHD prognosis.

## Introduction

Coronary heart disease (CHD) and depression are the two most common causes of disability.^1^ Compared with the general population, the prevalence of major depressive disorder (MDD) in patients with CHD is at least four times greater^2–4^; more than one-fifth of patients with CHD have depression; and up to one-third report elevated depressive symptoms.^2^ More rigorous and prospective data have demonstrated that MDD is an independent risk factor for CHD morbidity and mortality.^5 6^ In addition, a meta-analysis of prospective cohort studies (n=323 709) found that depression was associated with a 36% increase in the risk of coronary death (adjusted hazard ratio (HR)=1.36, 95% confidence interval (CI): 1.14 to 1.63) compared with non-depressed persons.^6^ Moreover, MDD incurs nervous system activation, cardiac rhythm disturbances, multidistrict immune and inflammatory responses, and hypercoagulability; notably, these changes negatively influence the cardiovascular system in CHD.^7^

Poor sleep quality and depression symptoms are related,^8 9^ with a higher prevalence of poor sleep quality in patients with cardiovascular diseases.^9^ Moreover, high prevalence rates of moderate-to-severe insomnia of 36%–37% have been reported during hospitalisation and 4–6 weeks after an acute cardiac event.^10^ Furthermore, sleep disturbances may increase the incidence of CHD.^11^ Insomnia symptoms are associated with a 45% increased risk of cardiovascular disease incidence or death from cardiovascular disease.^12^ Routine evaluations of sleep disturbance in CHD and further treatment allocation may contribute to reducing the long-term mortality associated with this disease.^13^

Despite evidence that MDD, poor sleep quality and heart disease are epidemiologically linked, this correlation is not well understood. MDD and poor sleep quality may exacerbate a common pathway, substantially elevating the risk of heart disease in individuals with both conditions. Immune inflammation may be a shared aetiological factor for mental disorders and CHD.^14^ Elevated inflammatory markers consistent with an acute-phase immunological response, especially C-reactive protein (CRP), interleukin-6 and tumour necrosis factor, are associated with MDD^15 16^ and an increased risk of cardiac morbidity and mortality.^17^ It has been suggested that antidepressants normalise proinflammatory states in depression and CHD.^18^ However, it is unclear whether depression is associated with poor sleep quality and cell-mediated immune function in patients with CHD. Therefore, questions remain regarding the relationship between MDD, poor sleep quality and cell-mediated functions in CHD.

Considering that MDD is associated with the complex pathophysiology of CHD, studying the inter-relationship between poor sleep quality and cell-mediated immune functions, as well as between inflammation and depression, appears to be a promising method. The present study aimed to investigate the contribution of depression to poor sleep quality and cell-mediated functions in CHD and to examine the discriminative factors in patients with CHD with and without MDD using subjective sleep quality and cell-mediated immune functions.

## Methods

### Procedure and subjects

**Figure 1:**
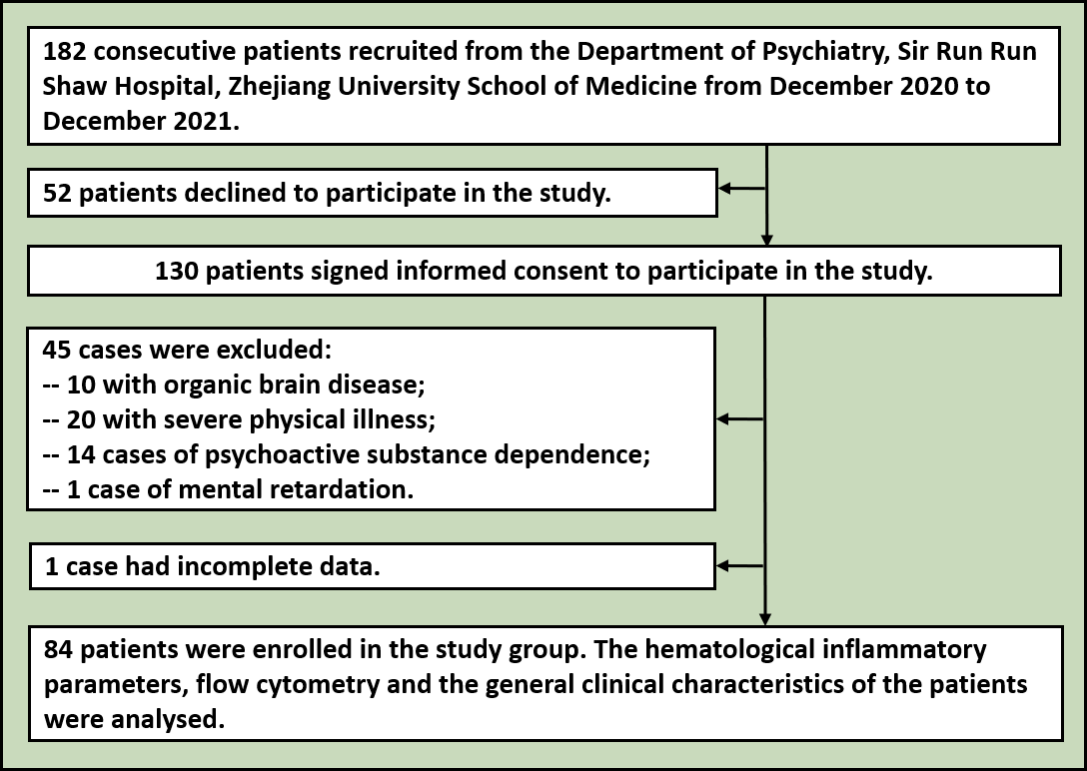
Flowchart of participants’ recruitment.

We used data from consecutive patients with MDD and CHD who attended the Department of Psychiatry and underwent a physical examination at Zhejiang University School of Medicine Sir Run Run Shaw Hospital between December 2020 and December 2021. They were evaluated according to our standard method, which includes the following criteria: (1) 18–80 years old; (2) provided written informed consent; (3) patients had evidence of CHD (previous hospitalisation for acute myocardial infarction, prior revascularisation procedure or coronary angiographic evidence of ≥50% blockage in one or more major coronary arteries) and met the diagnostic criteria for MDD according to the *Diagnostic and Statistical Manual of Mental Disorders*, Fifth Edition; (4) score on the Hamilton Depression Rating Scale (HAMD) of greater than 10 points^19^; and (5) no psychotropic medications used in the past month. Exclusion criteria were (1) autoimmune disease, severe systemic diseases, history of malignancies and/or treatment with chemotherapy, evidence of any concomitant inflammatory disease, acute infection, chronic inflammatory status or other acute cardiovascular diseases, as well as liver and kidney diseases; (2) history of glucocorticoid therapy within the past 3 months, secondary hypertension, heart failure and cerebrovascular disease; (3) history of dependence or abuse of alcohol, tobacco and other substance-related disorders; and (4) mental retardation or neurodevelopmental disorders, traumatic brain injuries and pregnancy. Of the 182 patients who met the initial inclusion criteria, 52 declined to participate and 130 provided informed consent. A total of 84 patients met the final inclusion criteria: 55 women and 29 men, of whom 50 had major depression (MDD+) and 34 had no major depression (MDD−) (figure 1).

## Measures

### Flow cytometry and blood routine tests

A 2 mL ethylenediamine tetraacetic acid (EDTA) sample of peripheral blood was obtained from each patient for flow cytometric analysis to analyse lymphocyte subsets. All tests were performed within a 4-hour sampling. Briefly, 25 µL of whole blood was mixed with 10 µL of mixed antibodies against a cluster of differentiation 3 (CD3), a cluster of differentiation 4 (CD4), a cluster of differentiation 8 (CD8), a cluster of differentiation 16 (CD16), a cluster of differentiation 19 (CD19) and a cluster of differentiation 56 (CD56) (Becton & Dickinson Bioscience, Multitest 6-Colour TBNK (lymphocyte subpopulation) reagent, 662967) and incubated in a dark room at 20°C–25°C for 15 min. Then, 450 µL of erythrocyte lysate was added to each tube and mixed well, and the tubes were incubated in a dark room at 20°C–25°C for 15 min. After centrifugation at 1500 rpm for 5 min, the supernatant was discarded and four to five drops of sheath solution were added to the machine. Lymphocyte subpopulations were detected by Becton & Dickinson Canto II, and data were analysed using Canto software. The total number of white blood cells (WBCs), the percentage of lymphocytes in WBCs and the absolute number of lymphocytes were determined by blood cell testing with Mindray BC-6900. The relative percentage and absolute count of each lymphocyte subset, including T cells, CD4^+^ T cells, CD8^+^ T cells, CD16^+^56^+^ natural killer (NK) cells and CD19^+^ B cells, were recorded, and the investigators were blinded to the clinical status of the study patients.

The CD45/side scatter (SSC) gating method was used for lymphocyte subsets, and the specific steps were as follows: (1) CD45/SSClow on the CD45/SSC scatter plot was set as the lymphocyte gate with CD45 as the horizontal axis and SCC as the vertical axis; (2) the absolute value and proportion of CD3^+^ T cells in lymphocytes were quantified; (3) the absolute value and proportion of CD3^+^CD4^+^ T cells and CD3^+^CD8^+^ T cells were measured; and (4) finally, the absolute value of CD19^+^ cells and the absolute value and proportion of B cells and CD16^+^CD56^+^ NK cells in lymphocytes were recorded.

Venous blood samples were drawn after an overnight fast of at least 8 hours. Inflammatory cells, including lymphocytes, monocytes, neutrophils and platelets, were acquired from routine blood tests, and the lymphocyte-to-monocyte ratio, neutrophil-to-lymphocyte ratio, Systemic Inflammation Index (SII) and platelet-to-lymphocyte ratio were evaluated.

### Assessment

The clinical symptoms were assessed by a team of trained psychometric specialists. After evaluating the five patients continuously, they demonstrated good reliability and consistency (average kappa >0.80). The HAMD had a reliability of 0.88–0.99 and a validity of 0.92. The Pittsburgh Sleep Quality Index (PSQI) had a reliability of 0.84–0.99 and a validity of 0.94.^20^

### Statistical analysis

The Shapiro-Wilk test was used to determine whether the data were normally distributed. Continuous data are expressed as the mean (SD). The chi-square test was used for data classification, while the Student’s t-test or the Mann-Whitney U test was used to compare parametric and non-parametric continuous variables, respectively. Relationships between the variables were ascertained using Pearson’s product moments. Multivariable analyses were performed using logistic regression models (forced entry) to adjust for HAMD and PSQI scores, sex, age and BMI. Linear discriminant analysis (LDA) was used to explore discriminative factors between groups. All results are expressed as arithmetic mean (SD). Statistical analyses were performed using SPSS V.22.0, and a p-value of ≤0.05 was considered statistically significant.

## Results

### Demographics and clinical characteristics

There were no significant differences in age, duration of illness, cardiac function or female:male ratio between the depressed and non-depressed groups. Compared with CHD without MDD, the PSQI scores were significantly higher in CHD with MDD (Z=−6.864, p<0.001; table 1).

**Table 1:** Comparison of demographic and clinical characteristics

Item	CHD MDD (+)	CHD MDD (−)	Statistics	P value
Age	58.46 (6.12)	57.68 (5.50)	Z=−0.762	0.441
Male:female ratio	18/32	11/23	χ^2^ *=*0.119	0.730
PSQI	12.36 (1.69)	8.06 (2.10)	Z=−6.864	<0.001
HAMD	19.98 (5.05)	9.15 (1.16)	Z=−7.734	<0.001
Age of illness onset	56.00 (7.88)	55.59 (6.79)	Z=−0.448	0.654
BMI	22.36 (2.30)	23.33 (2.25)	t=1.599	0.114
Cardiac function				
Resting heart rate	77.18 (7.05)	76.26 (7.82)	Z=−0.411	0.681
Systolic blood pressure	123.10 (6.64)	120.65 (6.09)	Z=−1.526	0.127
Diastolic blood pressure	78.30 (4.39)	76.82 (8.08)	Z=−0.361	0.718
Left ventricular ejection fraction	69.91 (5.36)	70.97 (4.06)	t=0.981	0.330

BMI, body mass index; CHD, coronary heart disease; HAMD, Hamilton Depression Rating Scale; MDD, major depressive disorder; PSQI, Pittsburgh Sleep Quality Index.

### Laboratory characteristics

As shown in table 2, SII (Z=−2.910, p*=*0.024) and CD4^+^/CD8^+^ T-cell ratio (Z=−3.249, p*=*0.001) were higher in patients with CHD with MDD than in those without MDD. CHD with MDD had fewer CD3^+^CD8^+^ T cells (Z=3.422, p*=*0.001) and CD3^+^ T cells (t=2.032, p*=*0.045) than those without MDD.

**Table 2:** Laboratory characteristics of the patient groups

Item	CHD MDD (+)	CHD MDD (−)	Statistics	P value
CD3^+^ T cell%	67.60 (7.08)	70.80 (7.16)	t=2.032	0.045
CD3^+^CD4^+^ T cell%	44.79 (6.40)	42.88 (6.34)	t=−1.344	0.183
CD3^+^CD8^+^ T cell%	19.77 (3.36)	24.27 (6.24)	Z=3.422	0.001
CD4^+^/CD8^+^ T-cell ratio	2.35 (0.60)	1.90 (0.60)	Z=−3.249	0.001
CD19^+^ B cell%	16.09 (4.52)	15.06 (5.62)	t=−0.891	0.376
CD16^+^56^+^ NK cell%	15.28 (6.90)	13.26 (6.52)	Z=−1.681	0.093
CD3^+^CD4^+^CD8^+^ T cell%	0.44 (0.49)	0.51 (0.68)	Z=0.447	0.665
CD3^+^ T-cell count, ×10^9^ /L	1.20 (0.38)	1.23 (0.46)	Z=0.410	0.682
CD3^+^CD4^+^ T-cell count, ×10^9^ /L	0.80 (0.29)	0.75 (0.27)	Z=−0.506	0.613
CD3^+^CD8^+^ T-cell count, ×10^9^ /L	0.35 (0.11)	0.43 (0.20)	Z=1.592	0.111
CD3^+^CD4^+^CD8^+^ T-cell count, ×10^9^ /L	0.01 (0.01)	0.01 (0.01)	Z=−1.506	0.132
CD19^+^ B-cell count, ×10^9^ /L	0.29 (0.13)	0.27 (0.14)	Z=−0.789	0.430
CD16^+^56^+^ NK-cell count, ×10^9^ /L	0.27 (0.15)	0.23 (0.13)	Z=−1.710	0.087
WBC count, ×10^9^ /L	6.16 (1.46)	5.73 (1.32)	Z=−1.591	0.112
NLR	2.27 (0.61)	2.00 (0.69)	t=−2.557	0.062
PLR	132.41 (46.50)	124.97 (34.57)	Z=−0.460	0.403
LMR	5.01 (1.19)	5.16 (1.87)	t=0.559	0.645
SII	502.88 (194.81)	419.13 (149.53)	Z=−2.910	0.024
CRP	1.42 (1.23)	1.10 (0.88)	Z=−1.393	0.164

CHD, coronary heart disease; CRP, C reactive protein; LMR, lymphocyte:monocyte ratio; MDD, major depressive disorder; NK cell, natural killer cell; NLR, neutrophil:lymphocyte ratio; PLR, platelet:lymphocyte ratio; SII, Systemic Inflammation Index; WBC, white blood cell.

### Assessment of the relationships between depression scores and sleep scores and immune cell levels in patients with CHD with MDD

**Figure 2:**
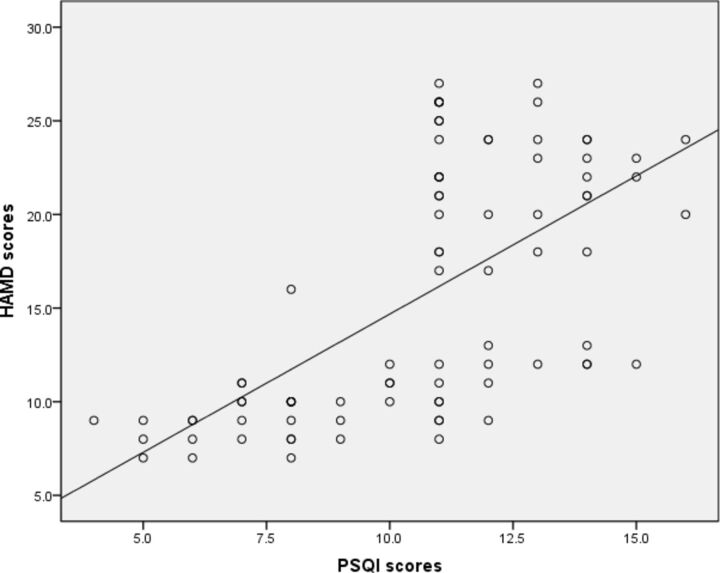
Scatter plot showing the distribution of HAMD scores by PSQI scores in depressed CHD. HAMD, Hamilton Depression Rating Scale; PSQI, Pittsburgh Sleep Quality Index.

Positive correlations were found between the HAMD scores and the CD4^+^/CD8^+^ T-cell ratio (r=0.571, p<0.001) and SII (r=0.464, p<0.001), PSQI global scores and SII (r=0.219, p=0.045), SII and the percentage of CD3^+^CD8^+^ T cells (r=0.310, p=0.004) in patients with CHD with MDD. The HAMD scores and the percentage of CD3^+^CD8^+^ T cells (r=−0.526, p<0.001), PSQI global scores and the percentage of CD3^+^CD8^+^ T cells (r=−0.272, p=0.012) were negatively correlated. HAMD scores were positively correlated with PSQI global scores (r=0.627, p<0.001) (figure 2). After simultaneous adjustment for HAMD scores, PSQI scores, sex, age and BMI, multivariate analyses showed that higher PSQI scores (OR=0.269, 95% CI: 0.144 to 0.500; p<0.001) were significantly associated with CHD and MDD. These results suggest that the severity of depression is correlated with abnormal T-cell levels and the PSQI in patients with CHD.

### Linear discriminant analysis

Table 3 shows the significant discriminant factors between groups. The LDA results were as follows: (1) pooled within-group correlation matrix not higher than 0.80; (2) pooled within-group covariance matrix Box’s M=2.959, approximate F=2.921, p=0.087; (3) functions at group centroids of 1.471 for depressed patients and −2.163 for non-depressed patients; (4) classification results demonstrated that 96.4% of the original grouped cases were correctly classified; and (5) eigenvalue=3.529, canonical correlation=0.875, Wilks’ lambda=0.235, χ^2^=114.473, df=6 and p<0.001. These findings suggest that patients with CHD with MDD may be differentiated from those without MDD according to the PSQI, SII and abnormal T-cell levels.

**Table 3:** Significant discriminant factors between depressed and non-depressed groups (linear discriminant analysis)

Variable	Wilks’ lambda	F	df	P value
PSQI	0.434	106.82	1–82	<0.001
HAMD	0.353	150.59	1–82	<0.001
SII	0.898	9.35	1–82	0.003
CD3^+^ T cell%	0.952	4.13	1–82	0.045
CD3^+^CD8^+^ T cell%	0.818	18.25	1–82	<0.001
CD4^+^/CD8^+^ T-cell ratio	0.880	11.18	1–82	0.001

HAMD, Hamilton Depression Rating Scale; PSQI, Pittsburgh Sleep Quality Index; SII, Systemic Inflammation Index.

## Discussion

### Main findings

The results of this cross-sectional study suggest that poor sleep quality, increased cell-mediated immunity and SII may predict depression in patients with CHD. These results have important implications for the recognition and treatment of comorbid depressive disorders in CHD. In this study, patients with CHD with MDD were characterised by a significantly higher PSQI score and elevated SII level, CD4^+^/CD8^+^ T-cell ratio and fewer CD3^+^CD8^+^ T cells and CD3^+^ T cells than patients without MDD. In addition, higher HAMD scores were correlated with the CD4^+^/CD8^+^ T-cell ratio, the percentage of CD3^+^CD8^+^ T cells, the percentage of CD3^+^ T cells, SII and PSQI scores in patients with CHD with MDD. In addition, the present study extends prior work^21^ linking depression, sleep quality, cell-mediated immune functions and CHD, demonstrating that poor sleep quality and abnormal T-cell levels may also be attributed to depression. Furthermore, patients with CHD with MDD may be differentiated from those without MDD by using these factors.

Patients with CHD with MDD also had a significantly higher PSQI score than those without MDD, suggesting that patients with CHD with MDD had poorer sleep quality than those without MDD and that ‘poor’ sleep quality is an important concomitant of CHD with MDD. This finding is consistent with that of a recent study.^9 10^ These findings support the hypothesis that emotional distress mediates the relationship between sleep disturbance and CHD mediated by emotional distress.^13 22^ In addition, it seems that having a higher HAMD score is associated with an increased probability of sleep disturbance in patients with CHD.^9^ Therefore, treatment of depression may offer the added benefit of diminishing sleep disturbances, thereby enhancing the quality of life of patients with CHD with depression.^13^

There was a positive correlation between the CD4^+^/CD8^+^ T-cell ratio, SII and HAMD scores in patients with CHD with MDD. The PSQI scores were also positively correlated with the SII. Furthermore, PSQI and HAMD scores were negatively correlated with the percentage of CD3^+^CD8^+^ T cells; therefore, higher HAMD and PSQI scores were associated with an increased probability of cell-mediated immune disturbance and inflammation.^23^ It has been well documented that depression and insomnia comorbidity is high in patients with systemic immunological diseases.^24^ On the other hand, previous studies have shown that sleep disturbances in individuals with depression are associated with impaired immune function and lower NK-cell activity, and significantly lower levels of immune cells (CD3^+^, CD4^+^ and CD8^+^)^25 26^ are associated with chronic insomnia. This suggests that depression may lead to sleep disturbances and abnormal cell-mediated immunity and inflammation, especially the percentage of CD3^+^CD8^+^ T cells, CD3^+^ T cells, the CD4^+^/CD8^+^ T-cell ratio and SII in patients with CHD. In addition, antagonism of the endogenous immune-inflammation process shows promise in improving depression and sleep quality and possibly in the remission of depression in groups of patients with high levels of inflammation.^27^ These findings are indirectly supported by reports that antidepressants may mediate some of the inflammation pathogenetic effects on reducing cardiovascular events.^19^

Furthermore, the CD4^+^/CD8^+^ T-cell ratio, the percentage of CD3^+^CD8^+^ T cells, the percentage of CD3^+^ T cells and SII were factors that discriminated between depressed and non-depressed patients. Some studies have demonstrated that cell-mediated immunity and inflammation, such as CD8^+^ T cells, CD4^+^/CD8^+^ T-cell ratio and SII, are increased in patients with depression.^28^ In addition, cell-mediated immunity is increased in patients with CHD.^27^ It was assumed that there is a relationship between sleep disturbance, cell-mediated immunity, inflammation and depression.^28^ These results suggest that T cells and SII are potential diagnostic biomarkers of CHD with depression.

Overall, the present study results highlight that depressive symptoms are a potentially important risk factor for CHD and suggest that depression may be strongly associated with the risk of insomnia and abnormal T-cell levels in CHD.^29^ However, depression may also contribute to the underlying disease process by disrupting sleep quality and cell-mediated immunity in CHD.^28^

### Limitations

Although our sample size was comparable to those of other studies, it was relatively small; therefore, larger studies should be conducted to determine whether antidepressants may further improve sleep quality, cell-mediated immunity and inflammation in depressed CHD. The patients were not followed up to explore the relationship between the course of depression and T-cell levels. Additionally, this study lacked a healthy control group and/or a group with other psychiatric disorders as comparators.

### Implications

In conclusion, there are close relationships between major depression and elevated SII, poor sleep quality and increased cell-mediated immunity, especially CD3^+^ T cells, CD3^+^CD8^+^ T cells and the CD4^+^/CD8^+^ ratio in patients with CHD and depression, possibly leading to elevated SII, poor subjective sleep quality and abnormal T-cell levels in CHD. Furthermore, these variables classified patients with and without depression with an accuracy of 96.4%. The study results may inform potential strategies for the screening, prevention or treatment of MDD in patients with CHD. Clinicians should be aware of these identified discriminative factors because treating insomnia and depression may also improve CHD prognosis.

## Data Availability

Data are available upon reasonable request.
